# Use of Non-Conventional Cell Disruption Method for Extraction of Proteins from Black Yeasts

**DOI:** 10.3389/fbioe.2016.00033

**Published:** 2016-04-15

**Authors:** Maja Čolnik, Mateja Primožič, Željko Knez, Maja Leitgeb

**Affiliations:** ^1^Laboratory for Separation Processes and Product Design, Faculty of Chemistry and Chemical Engineering, University of Maribor, Maribor, Slovenia

**Keywords:** *P. triangularis*, *W. ichthyophaga*, *T. salinum*, supercritical carbon dioxide, enzyme activity, cell disruption

## Abstract

The influence of pressure and treatment time on cells disruption of different black yeasts and on activities of extracted proteins using supercritical carbon dioxide process was studied. The cells of three different black yeasts *Phaeotheca triangularis, Trimatostroma salinum*, and *Wallemia ichthyophaga* were exposed to supercritical carbon dioxide (SC CO_2_) by varying pressure at fixed temperature (35°C). The black yeasts cell walls were disrupted, and the content of the cells was spilled into the liquid medium. The impact of SC CO_2_ conditions on secretion of enzymes and proteins from black yeast cells suspension was studied. The residual activity of the enzymes cellulase, β-glucosidase, α-amylase, and protease was studied by enzymatic assay. The viability of black yeast cells was determined by measuring the optical density of the cell suspension at 600 nm. The total protein concentration in the suspension was determined on UV–Vis spectrophotometer at 595 nm. The release of intracellular and extracellular products from black yeast cells was achieved. Also, the observation by an environmental scanning electron microscopy shows major morphological changes with SC CO_2_-treated cells. The advantages of the proposed method are in a simple use, which is also possible for heat-sensitive materials on one hand and on the other hand integration of the extraction of enzymes and their use in biocatalytical reactions.

## Introduction

The widespread use of fungi in different biotechnological processes can be attributed to their intrinsic characteristics. They can secrete a wide range of extracellular enzymes and proteins, which represents a powerful biochemical tool for the catalysis of a number of valuable reactions (i.e., regio- and stereo-selective reactions). A lot of biological molecules are inside the cell, and they must be released from it. This could be achieved by cell disruption (lysis). Cell disruption is a sensitive process because of the cell wall’s resistance to the high osmotic pressure inside them. Furthermore, difficulties arise from a non-controlled cell disruption that results from an unhindered release of all intracellular products (proteins nucleic acids, and cell debris) as well as the requirements for cell disruption without the desired product’s denaturation.

The large amounts of toxic solvents and energy required make the conventional disruption methods unattractive. Therefore, the development of an efficient recovery method is important. Supercritical fluids have unique physicochemical properties such as high densities and low viscosities and that make them suitable as extraction solvents. Carbon dioxide (CO_2_) is the most beneficial SCF used in extraction processes. SC CO_2_ is non-flammability, non-toxicity, as well as the selectivity of the process, and the ease of recovery are the most important features of its usage (Darani and Mozafari, 2009; Mun et al., 2011). SC CO_2_ is the most popular used SCF, since it is easy to use and it is inexpensive solvent. SC CO_2_ treatment is an alternative method for the inactivation of microorganisms. The higher the temperature and pressure of the SC CO_2_, the higher the percentage of the inactivated microorganisms. At higher pressures, the solubility of CO_2_ in water increases, which allows the formation of carbonic acid. Higher temperatures stimulate the diffusion of CO_2_. Fluidity of the membrane increases, and consequently to improve the excretion of cellular material from the cells (Garcia-Gonzalez et al., 2007; Wimmer and Zarevúcka, 2010; Mun et al., 2011; Ji et al., 2012). Generally, treating microorganisms with SC CO_2_ at higher pressure or temperature, or treating them for a longer time results in greater microbial reduction than a treatment at lower pressure or temperature, or treating them for shorter times (Wimmer and Zarevúcka, 2010). However, at too high temperature, enzyme deactivation can occur. The use of SC CO_2_ for the inactivation of microorganisms can be safely used in foods and bioactive substances at relatively low temperatures (Debs-Louka et al., 1999; Hong and Pyun, 2001; Erkmen, 2003; Wimmer and Zarevúcka, 2010; Ortuño et al., 2012). However, SC CO_2_ can also serve as a solvent for the extraction of intracellular components from microbial cells or for isolation of products from the reaction mixture in the production of biomass (Spilimbergo et al., 2003; Wimmer and Zarevúcka, 2010; Ceni et al., 2016).

The potential application of some organisms is often determined by their taxonomic and phylogenetic diversity, as well as their occurrence and adaptation to various habitats (Raghukumar, 2012). Salinity is the most defining feature of the oceanic environment. Salt pans are an attractive source for extremophilic microorganisms (Raghukumar, 2012). No obligate marine fungi have been detected in salt pans till now, but they present a suitable environment for fungi, such as black yeasts, *Trimatostroma salinum, Hortaea werneckii*, and *Pheaotheca triangularis*, which were isolated from salt pans of 15–30% salinity (Zalar et al., 1999; Gunde-Cimerman et al., 2000). Black yeast is a term subscribing a group of fungi that is quite heterogeneous from the taxonomy and phylogenetic point of view but having in common melanized cell walls and the formation of daughter cells by yeast-like multilateral or polar budding (Rosa and Peter, 2006). Black yeasts have a unique ability to survive where other microorganisms are rarely observed. Their name comes from the fact they produce the dark pigment melanin, unlike other yeasts. Melanin is the expression of the dark brown to black pigments phenol compounds formed by oxidative polymerization and assemblies of proteins and carbohydrates (Sterflinger, 2006; Kutty, 2009). The proteins of halophilic archaea are highly adapted to function at high ionic strength. Because of this extreme saline environment, halophilic proteins represent a valuable resource for understanding the processes of natural selection and adaptive evolution.

The study was focused on the possibility to obtain the active form of cellulase, α-amylase, β-glucosidase, and protease after destruction of black yeast cell walls with SC CO_2_. For comparison, the cell wall could be broken using different mechanical methods, e.g., the homogenization, sonication, and bead milling. Homogenization is an efficient way to apply shearing forces to break down cell wall of microorganisms. It may be used to disrupt bacteria and yeast cells at the laboratory and industrial scale. The disadvantage of the valve homogenizers when applied to extraction of heat sensitive materials is the need for external cooling, which can only be applied after the disruption (with the concomitant temperature increase) has taken place (Geciova et al., 2002). Sonication is a mechanical method where cells are broken by sonic cavitation. Sonication is very easy to adjust by amplitude setting, a sonication time, as well as by choosing the right equipment; it is possible to disrupt cell membranes very gently or very abruptly, depending on the cell structure. The heat, generated by the ultrasound process, must be dissipated, and high noise levels appear. Therefore, the use of this method is very expensive. Significant degradation of enzymes due to heat denaturation may occurred because of insufficient cooling in close proximity of the sonication probe (Geciova et al., 2002). The bead mill provides a simple and effective way for disruption of different types of microorganisms. The degree of disruption increases with bead loading due to increased bead-to-bead interaction. Consequently, heating and power consumption also increase. The enzymes found in soluble form in the cytoplasm are released with higher efficiency using smaller beads. For enzymes bound to cytoplasmic membrane or in periplasmic space, bigger glass beads can be used (Geciova et al., 2002). This method has many disadvantages such as uneven processing, protein denaturation, low efficiency while relatively high energy consumption, complex separation of milling medium, and product- and time-consuming cleaning. Although the mechanical methods are favored due to economic advantages, SCF usage has significant advantages (described previously) to use them for cell disruption (Darani and Mozafari, 2009). Usage of SC CO_2_ in biocatalysis has an important preference; it can act as a medium for opening the cells and carrying out the biocatalytical reaction at the same time.

The impact of different experimental conditions (pressure and incubation time) on viability of cells, total protein concentrations, and cellulase, α-amylase, β-glucosidase, and protease activities in the suspensions of black yeasts during SC CO_2_ treatment was investigated.

## Materials and Methods

### Materials

*Pheaotheca triangularis* EXF-206, *T. salinum* EXF-295, and *Wallemia ichthyophaga* EXF-5676 were obtained from the University of Ljubljana, Biotechnical Faculty, Department of Biology (Ljubljana, Slovenia). Carbon dioxide 2.5 (purity 99.5%) was supplied by Messer MG (Ruše, Slovenia). Peptone from meat, potassium phosphate, potassium dihydrogen phosphate, sodium carbonate, sodium bicarbonate, and acetic acid were purchased from Merck (Darmstadt, Germany). Sodium pyrophosphate decahydrate (≥99.0%), sodium phosphate monobasic (≥99.0%), sodium phosphate dibasic (≥99.0%), albumin from bovine serum (BSA) (≥98.0%), malt extract, agar, d-(+)-glucose, sodium acetate, Sigmacell, glucose assay reagent, Casein, Hammarsten bovine, trichloroacetic acid (TCA), d-(−)-Salicin (≥99.0%), starch azure, and sodium chloride were supplied from Sigma (Schnelldorf, Germany).

### Preparation of Black Yeasts Suspension

The microbial strains used in this study were *P. triangularis, T. salinum*, and *W. ichthyophaga*. *P. triangularis* and *T. salinum* grown on malt extract agar (MEA) for 5–7 days at room temperature, while cells of *W. ichthyophaga* grown on MEA 17% NaCl at the same condition. Each of black yeast cells were suspended to sterile saline solutions. The cultures used in all experiments were freshly prepared by the same procedure.

### Treatment of Black Yeasts with SC CO_2_

**Figure 1:**
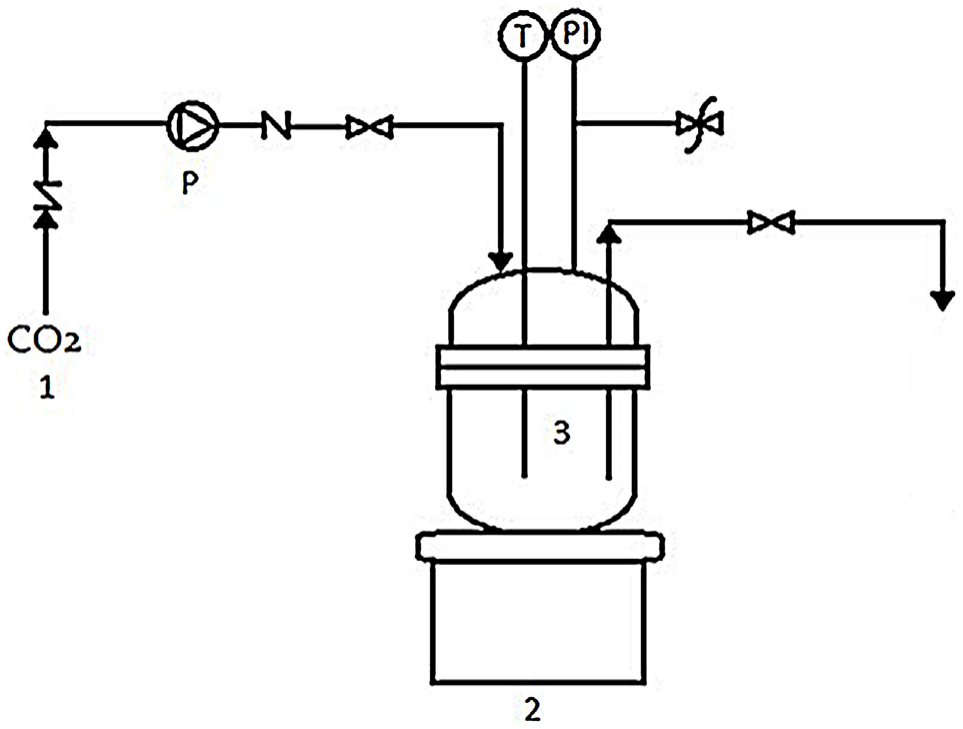
**High-pressure batch reactor: (1) CO_2_ tank, (2) heater, (3) high-pressure batch reactor, (P) high-pressure pump, (T) temperature regulator, and (PI) pressure indicator**.

Experiments were carried out in a 70-mL high-pressure batch reactor (Figure 1). The sterile ampoule was filled with freshly prepared cell suspension of black yeast and placed into the reactor. The reactor was tightly closed and immersed in an oil bath at the fixed temperature of 35°C. Next, the reactor was charged to the desired pressure of 10 or 30 MPa with cooled CO_2_. After the cell suspension of black yeast was exposed to SC CO_2_ for a certain time, the pressure was released slowly (Δ*p*/Δ*t* = 0.5 MPa/min) to atmospheric pressure. The culture broth in ampule was immediately removed from the reactor and transferred into the sterile tube, for the subsequent analyses. The reactor was rinsed with distilled water and autoclaved.

### Determination of Enzymatic Activities

The optical densities of black yeast cells in a suspension before incubation in SC CO_2_ were between 0.48 and 0.52. Before further analysis, each of the cell suspensions of black yeasts was centrifuged for 2 min after incubation in SC CO_2_. The supernatant without cells and debris was analyzed spectrophotometrically.

The amount of total proteins was determined with Bradford method (Bradford, 1976). Activities of the cellular enzymes of black yeasts, α-amylase, cellulase, β-glucosidase, and protease, which are the most commonly used enzymes in applied biocatalysis, were determined by the UV–Vis spectrophotometer at wavelengths of 595, 340, and 280 nm before and after incubation of the black yeasts culture in SC CO_2_. pH of black yeasts suspensions was measured with a pH meter (Hanna instruments) before and after incubation in SC CO_2_. The optical density of black yeast cell suspensions was determined at 600 nm by the UV–Vis spectrophotometer.

#### Cellulase Activity Assay

Sigmacell solution (4 mL, 5% *w*/*v*) was added to 1 mL of cell suspension of studied culture and mixed at 37°C for 120 min with moderate shaking. After that, the suspension was immediately transferred into an ice bath. After the suspension was settled, it has been centrifuged for 2 min to clarify. To 3 mL of a substrate solution, 100 μL of the supernatant was added. The increase of absorbance at 340 nm followed at 25°C with a UV–Vis spectrophotometer was used to define the cellulase activity.

#### α-Amylase Activity Assay

Amylose azure (90 mg) was dissolved in 9 mL of potassium phosphate buffer, and the suspension was incubated at 37°C. Then, 0.5 mL of studied culture cell suspension was added, mixed by swirling, and placed in a shaker bath at 37°C for 10 min. Acetic acid solution (0.5 mL) was poured into the reaction mixture. The resulting mixture was filtered. The increase of absorbance at 595 nm was used to define the α-amylase activity. All absorbance measurements were performed with a UV–Vis spectrophotometer. The activity was expressed as the change in absorbance at 595 nm per minute per solid of α-amylase.

#### Protease Activity Assay

Casein solution (1 mL) was added to previously prepared phosphate buffer. Solution was stirred and heated up to 35°C. Then, 0.5 mL of phosphate buffer and 0.5 mL of selected black yeast cell suspension were pipetted and incubated at 35°C for 20 min. After incubation, 3 mL of TCA was added and incubated at room temperature for 30 min. Thus, prepared reaction mixture was centrifuged at 3000 rpm for 20 min. Supernatant was measured at 280 nm on UV–Vis spectrophotometer.

#### β-Glucosidase Activity Assay

Salicin solution (2 mL) was pipetted into centrifuge falcon tubes and incubated at 37°C for exactly 10 min. Then, 2.5 mL of carbonate-bicarbonate buffer solution was added. This stopped reaction mixture was used in next step. To 0.9 mL of a glucose reagent solution, 100 μL cell suspension of selected black yeast was added, mixed by inversion, and the reaction was monitored at 340 nm with a UV–Vis spectrophotometer.

## Results and Discussion

### Viability of Black Yeast Cells Exposed to SC CO_2_

*Wallemia ichthyophaga*, *T. salinum*, and *P. triangularis* type strains were isolated from the hypersaline water of the Sečovlje solar saltern. *W. ichthyophaga* requires growth media with reduced water activity (*a*_w_) (0.959–0.771). It grows better on media with high concentration of NaCl (Zalar et al., 2005a). It is a fungus from the genus *Wallemia* (*Wallemiales*, *Wallemiomycetes*) that grows only at salinities between 10% (*w*/*v*) NaCl and 32% (*w*/*v*) saturated NaCl solution (Zajc et al., 2014). Therefore, *W. ichthyophaga* is the most halophilic fungus known to date (Zajc et al., 2014). *T. salinum* is a halophilic constitutively melanized fungi, which was isolated from hypersaline water at the highest salinity level during the season of salt production from Adriatic Sea. *T. salinum* was found also on mats and wood, immersed in brine, and is able to decompose wood at hypersaline conditions (Kogej et al., 2006). *P. triangularis* shows high adaptability to saline conditions, with the highest frequency of appearance in water between 22 and 28% salt concentration. *P. triangularis* was unable to grow on 32% salt and 26% NaCl being the maximum (Gunde-Cimerman et al., 2000).

Two most common reasons for cell death are either structural damage (disruption of the envelopes, DNA conformational changes, ribosome alterations, or protein aggregation) or physiological dysfunctions (membrane selective permeability alterations or loss of function of key enzymes) (Manas and Pagan, 2005).

**Figure 2:**
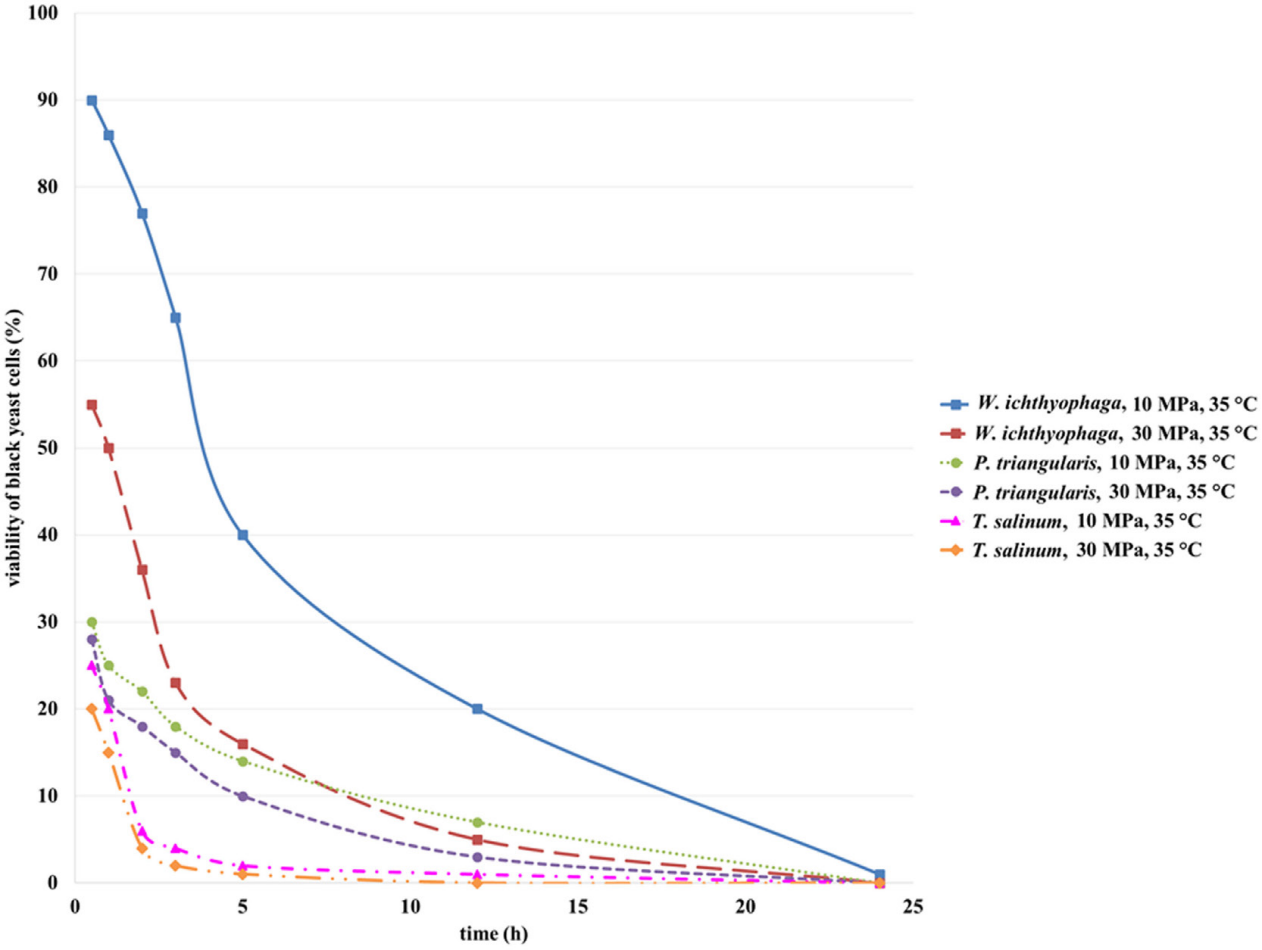
**Viability of *W. ichthyophaga, P. triangularis*, and *T. salinum* cells after incubation of cell suspensions in SC CO_2_ at 10 and 30 MPa and 35°C versus incubation time**.

Viability of black yeast cells, *W. ichthyophaga, P. triangularis*, and *T. salinum*, in suspension culture after SC CO_2_ treatment was determined. Selected black yeast cell suspension (1 mL) after treatment in SC CO_2_ was added in the sterile prepared meat extract broth and incubated at 25°C for growth. Viability of selected black yeast cells was monitored spectrophotometrically by measuring optical density of the culture broth at 600 nm. Figure 2 shows that the viability of all studied cell cultures after 1 h incubation in SC CO_2_ at 10 and 30 MPa decreased. Cell viability decreased as the treatment time and pressure increased. SC CO_2_ treatment decreased pH of the black yeast cell suspension from 5.9 to 4.5 because of the acidification of CO_2_ in the aqueous medium. The highest viability (90%) after incubation in SC CO_2_ was observed for the cell suspension of *W. ichthyophaga* at 10 MPa, 35°C, and incubation time of 30 min. Quite high viability of black yeast *W. ichthyophaga* (55%) was also observed after incubation in SC CO_2_ for 30 min at 30 MPa and 35°C. The viability of cells in *P. triangularis* cell suspension after 30 min incubation in SC CO_2_ at 10 MPa and 35°C was about 30%. The lowest viability of cells (20%) after 30 min exposure to SC CO_2_ was achieved in *T. salinum* cell suspension at 30 MPa and 35°C. In all cases, no survival of cells after 24 h of incubation in SC CO_2_ was detected.

Based on these results, we can conclude that the cells of black yeast *W. ichthyophaga* are quite resistant to elevated pressure. Since, the *W. ichthyophaga* is known as the most halophilic fungus known to date (Zajc et al., 2014), the high viability of its cells exposed to high pressure CO_2_ could be attributed to its endurance under extreme conditions.

**Figure 3:**
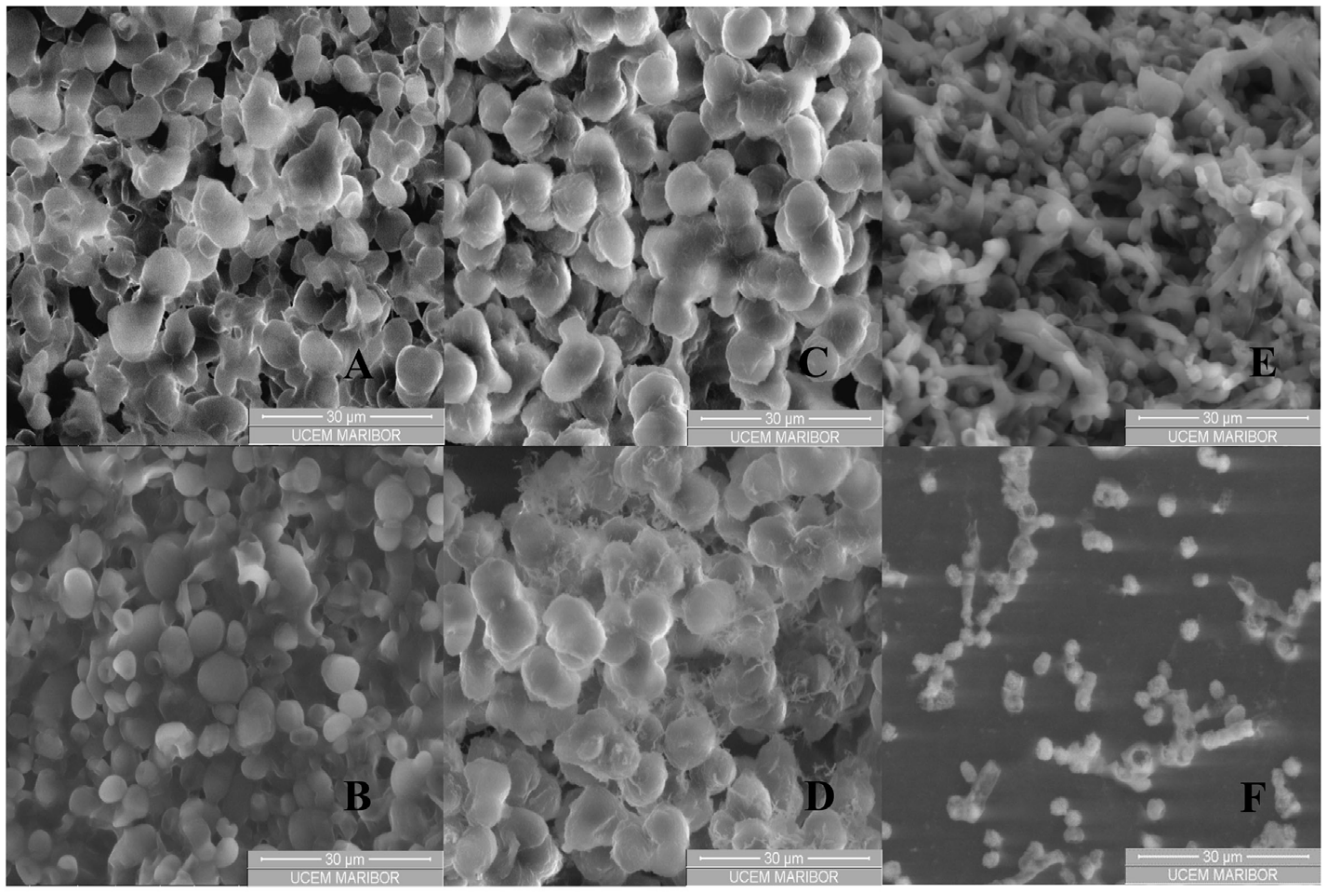
**ESEM images of black yeast cell suspension before and after incubation in SC CO_2_**. **(A)** *P. triangularis* cells before incubation in SC CO_2_, **(B)** *P. triangularis* cells after 24 h incubation in SC CO_2_ at 30 MPa and 35°C, **(C)** *T. salinum* cells before incubation in SC CO_2_, **(D)** *T. salinum* cells after 24 h incubation in SC CO_2_ at 30 MPa and 35°C, **(E)** *W. ichthyophaga* cells before incubation in SC CO_2_, and **(F)** *W. ichthyophaga* cells after 24 h incubation in SC CO_2_ at 30 MPa and 35°C.

Black yeast cells were observed under an environmental scanning electron microscope (ESEM) at different magnifications before and after incubation of cell suspensions in SC CO_2_ (Figures 3A–F). SC CO_2_ has a significant impact on black yeast cells. As could be evident from Figures 3B,D,F, the cells exposed to SC CO_2_ were very damaged and deformed. *P. triangularis*, *T. salinum*, and *W. ichthyophaga* cells were burst after exposed to SC CO_2_ and they lost their shape.

Supercritical fluid cell disruption is a process, wherein a sudden release of the applied SC CO_2_ pressure results in its penetration into the cells. After expansion of gas within the cells, discharge of pressure damages the cell wall and consequently the cell disruption occurred (Darani and Mozafari, 2009). Higher pressure leads to inactivation of *P. triangularis, T. salinum*, and *W. ichthyophaga* cells. Viability of the cells was influenced by the physical properties of CO_2_, such as density and viscosity which were higher at 30 MPa and 35°C, compared to the physical properties of CO_2_ at 10 MPa and 35°C (Table S1 in Supplementary Material). After 24 h incubation of cell suspensions in SC CO_2_, almost all cells of *P. triangularis*, *T. salinum*, and *W. ichthyophaga* were dead (Figures 3B,D,F).

### Residual Concentration of Total Proteins in Suspension of Selected Black Yeast after Treatment with SC CO_2_

Residual concentration of proteins in cell suspension of the selected black yeast was determined by Bradford assay. The Bradford assay is fairly accurate, and samples that are out of range can be retested within minutes (Bradford, 1976). It is recommended for general use, especially for determining protein content of cell fractions and assessing protein concentrations for gel electrophoresis (Karthikeyan and Santosh, 2009).

The treated cell suspension of selected black yeast was centrifuged at 11,000 rpm. Supernatant was used for analysis.

**Figure 4:**
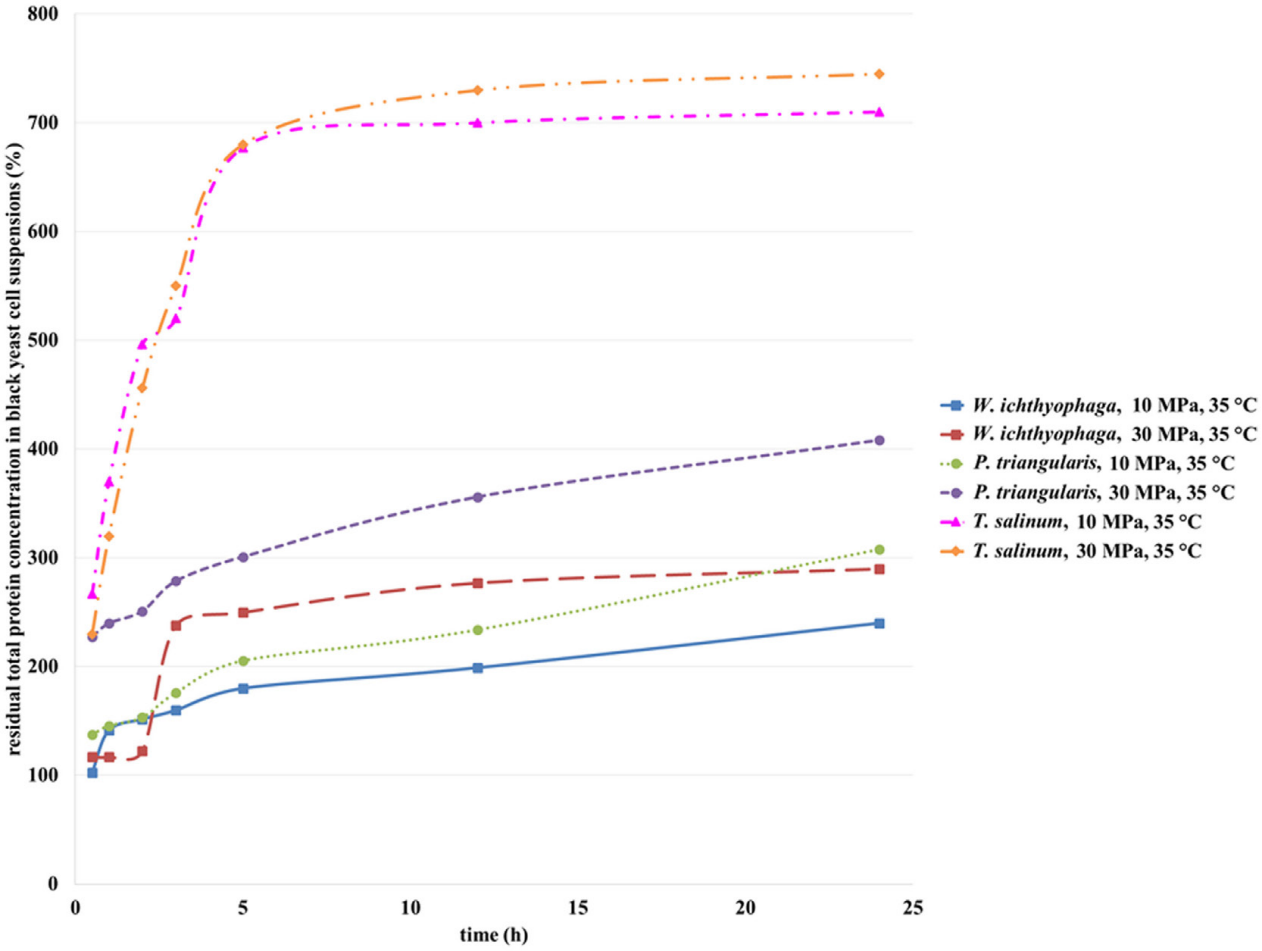
**Residual total protein concentration in cell suspensions of *W. ichthyophaga, P. triangularis*, and *T. salinum* after incubation in SC CO_2_ at 10 and 30 MPa and at 35°C versus incubation time**.

Total residual protein concentration (Figure 4) for all studied cell suspensions increased with the increase of incubation time in SC CO_2_. SC CO_2_ effects the black yeast cells by damaging the cell wall, and intracellular material can be removed from the cell and extracted in cell suspension. Consequently, eliminated intracellular proteins influence total protein concentration in cell suspensions of the studied black yeasts. Inside the black yeast cells free or on cellular structures bound enzymes are presented (intracellular enzymes). Besides, some excreted enzymes in the growth medium (extracellular enzymes) could be found. The highest residual protein concentration in the cell suspension of *T. salinum* was detected after 24 h of incubation in SC CO_2_ at 30 MPa, 35°C (Figure 4). Residual protein concentrations in the cell suspensions of *W. ichthyophaga* and *P. triangularis* after incubation in SC CO_2_ increased, but were lower than those in the case of *T. salinum* regardless of the increase in pressure. Generally, at higher pressure, higher residual protein concentrations in all studied cell suspensions of black yeasts were detected. This coincides with the findings from the previous study of viability. The lowest residual protein concentration at both tested pressures was detected in *W. ichthyophaga* cell suspension, which is conditioned by the fact that for same tested culture, the highest cell viability at same conditions was determined. At lower cell viability, due to inactivation and burst of the cell, more proteins were extracted from the cell and consequently higher residual protein concentration was detected, as it was the case in *T. salinum* cell suspension.

Mun et al. (2011) published about a high increase in insoluble protein concentration of *Pseudomonas aeruginosa* cell suspension after SC CO_2_ treatment what may reflect the protein denaturation inside the cells because of the acidification during this process. This acidification may have led to the chemical and physical modification of the lipid bilayer of the membrane, thus causing disorder in the intracellular metabolic balance or removal of the vital constituents from cells. For this reason, activities of some enzymes in black yeast cell suspensions after exposure to the SC CO_2_ were studied.

### Activities of Cellulases, α-Amylases, β-Glucosidases, and Proteases from Black Yeast Cell Suspensions

#### Residual Activities of Cellulases

Microbial cellulases have become the focal biocatalysts due to their complex nature and wide spread industrial applications. Cellulases are composed of independently folding, structurally and functionally discrete units called domains or modules, making cellulases module. Cellulases are inducible enzymes synthesized by a large diversity of microorganisms including both fungi and bacteria during their growth on cellulosic materials. These microorganisms can be aerobic, anaerobic, mesophilic, or thermophilic. Among them, the genera of *Clostridium*, *Cellulomonas*, *Thermomonospora*, *Trichoderma*, and *Aspergillus* are the most extensively studied cellulase producer (Kuhad et al., 2011).

**Figure 5:**
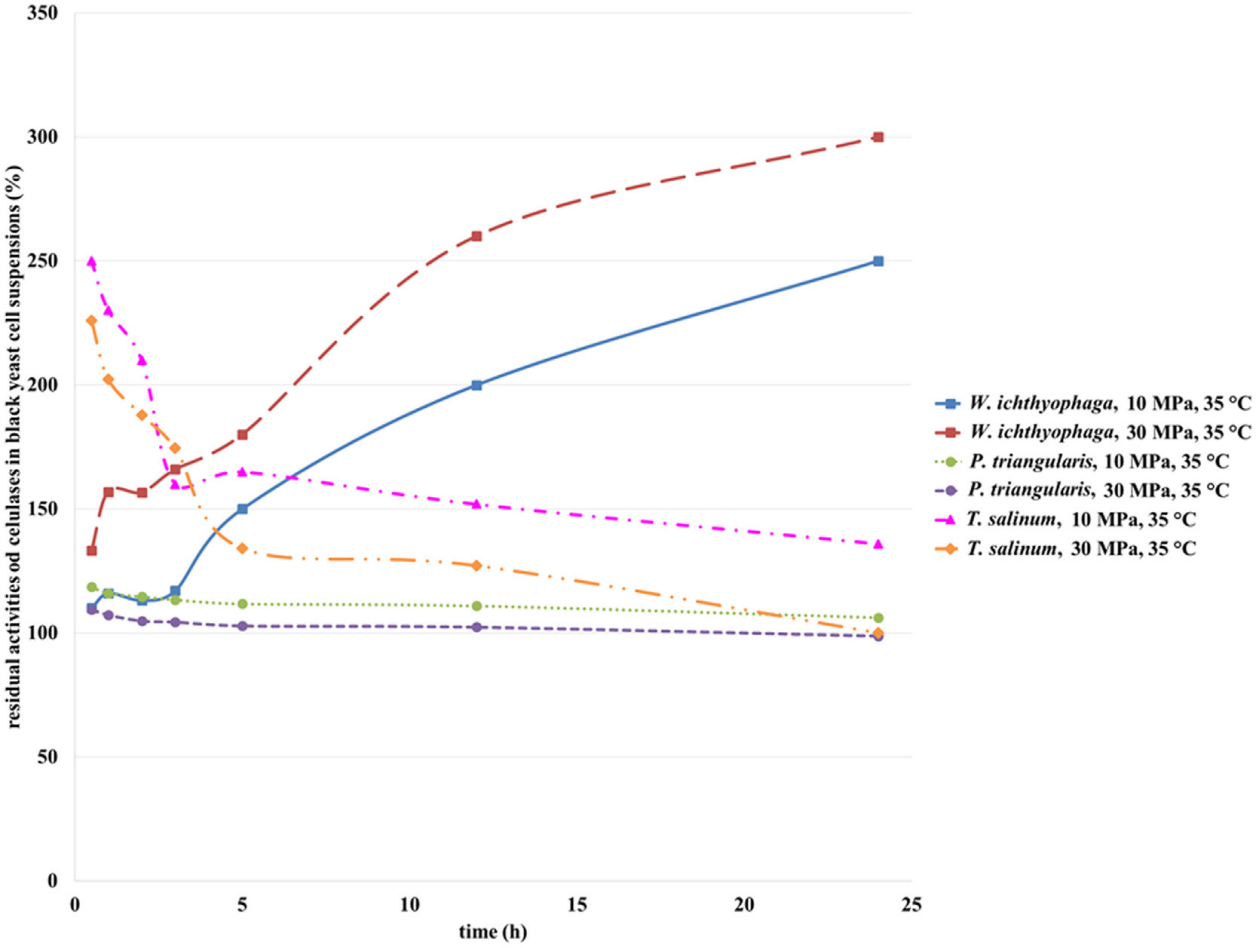
**Residual activities of cellulases from *W. ichthyophaga, P. triangularis*, and *T. salinum* after incubation in SC CO_2_ at 10 and 30 MPa and at 35°C as a function of incubation time**.

Cellulase is an extracellular enzyme of *T. salinum* (Zalar et al., 2005b) and an intracellular enzyme of *P. triangularis* and *W. ichthyophaga* (personal communication, Zalar and Gunde-Cimerman, 2010), which activities were determined in the cell suspensions of studied cultures before and after incubation in SC CO_2_. The activity of cellulase from black yeast cells before incubation in SC CO_2_ was defined as 100%. Figure 5 shows the residual activity of cellulases in black yeast suspensions after incubation in SC CO_2_. After exposure of *T. salinum* and *P. triangularis* cell suspensions in SC CO_2_ (Figure 5), the cellulase activities started to decrease as a function of incubation time. The reason for such behavior is probably due to enzyme deactivation at both examined pressures (10 and 30 MPa). It is well known that the activity of enzymes exposed to CO_2_ under high-pressure depend on enzyme species, water content in the solution, and the pressure and temperature of the reaction system. The three-dimensional structure of enzyme may be significantly altered under extreme conditions, causing their denaturation and consequent loss activity (Wimmer and Zarevúcka, 2010). Since the cellulase is an extracellular enzyme of *T. salinum*, it is more exposed to the extreme conditions (high pressure, acidification during the SC CO_2_ treatment), than in the case of *P. triangularis* and *W. ichthyophaga*, where the cellulase is an intracellular enzyme, and it is more protected from the effects of supercritical medium.

From Figure 5, it could be seen that the activity of cellulase from *W. ichthyophaga* exposed to SC CO_2_ at both examined pressures increased as a function of incubation time in SC CO_2_. The maximum residual activity of cellulase from *W. ichthyophaga* cell suspension in the SC CO_2_ was reached after 24 h exposure at 30 MPa, 35°C and was amounted to 300%. This can be attributed to a large quantity of extracted cellulase from the cells of *W. ichthyophaga*. Obviously, the cells of *W. ichthyophaga* contain a large amount of cellulase. No significant difference in cellulase activity from *P. triangularis* at both studied pressures in dependence of incubation time was detected. The reason for this could be in low cellulase concentration in *P. triangularis* cells.

It is evidenced that the pressure influenced the activity of cellulase from *T. salinum*, where with increase in pressure, the decrease in cellulase activities were determined. Because the cellulase from *T. salinum* is an extracellular enzyme, the exposure to SC CO_2_ could be the reason for its fast deactivation.

#### Residual Activities of α-Amylases

α-Amylase can be isolated from plants, animals, or microorganisms. It is produced by many bacteria. The most frequently used source for the commercial production of the α-amylases are *Bacillus amyloliquefaciens* and *Bacillus licheniformis* (de Souza and de Oliveira Magalhães, 2010; Sundarram and Thirupathihalli, 2014).

Enzymes produced from these microorganisms show promising prospects in many industrial applications, such as food, fermentation, textile, and paper industries. *Bacillus licheniformis* produces thermostable α-amylase, which is of most importance, since the hydrolysis of the starch is carried out at high temperatures. Thermostable enzymes were studied in order to improve industrial processes of starch degradation and are useful for the production of valuable products, such as glucose, maltose, and dextrose. Sources of α-amylase from fungi are mainly limited to isolates of *Aspergillus* species (Senyay-Oncel and Yesil-Celiktas, 2011) and some species of *Penicillium* (Sundarram and Thirupathihalli, 2014).

**Figure 6:**
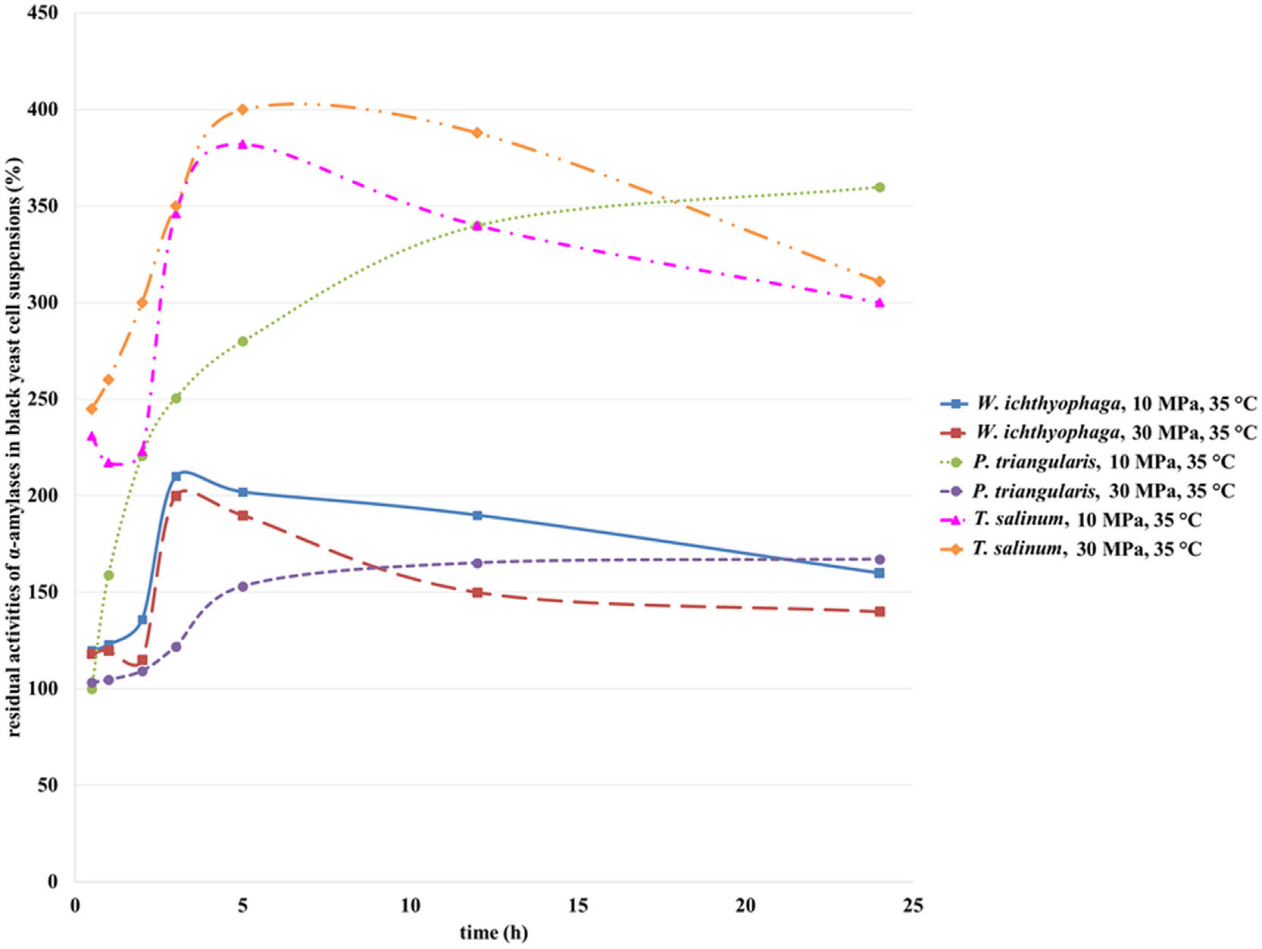
**Residual activities of α-amylases from *W. ichthyophaga*, *P. triangularis*, and *T. salinum* after incubation in SC CO_2_ at 10 and 30 MPa and at 35°C as a function of incubation time**.

α-Amylases from *T. salinum* (Zalar et al., 2005b), *W. ichtyhyphaga*, and *P. triangularis* are intracellular enzymes (personal communication, Zalar and Gunde-Cimerman, 2010). The activity of α-amylase from black yeast cells before incubation in SC CO_2_ was defined as 100%. After incubation of the cell suspensions in SC CO_2_, the α-amylase activity was increased due to opening of the *T. salinum* and *W. ichtyhyphaga* cells and the elimination of the enzyme in the medium. The activity of α-amylase from *T. salinum* increased during the first 5 h of incubation in SC CO_2_ at both studied pressures (Figure 6), but with longer incubation time, the α-amylases activity decreased. The highest residual activity of α-amylase in *T. salinum* cell suspension was achieved after 5 h of incubation in SC CO_2_ at 30 MPa, 35°C (Figure 6) and was detected to be 400%. At this incubation time, the most of the cells were opened and the enzyme was released in the cell suspension. With higher incubation time, α-amylase was deactivated due to the prolonged contact with SC CO_2_. When the *W. ichityophaga* cell suspension was exposed to SC CO_2_, increase in residual activity of α-amylase was detected during first 3 h of incubation in SC CO_2_. With further increase in incubation time, a decrease in residual activity of α-amylase at both studied pressures was detected. Higher residual activities were determined at lower studied pressure, which indicates the fact that higher pressure has a negative influence on the activity of intracellular α-amylase. The reason for such activity loss could be due to high pressure, which could cause deactivation of enzymes or most probably due to the change in pH of cell suspension owing to formation of carbonic acid by the contact of water with pressurized CO_2_.

Residual activity of α-amylase from *P. triangularis* increased with the increase of incubation time regardless of the selected pressure. Higher residual activity of α-amylase was detected at lower pressure (10 MPa). Since α-amylase from *P. triangularis* is an intracellular enzyme, the increase in its activity is connected with the cell opening due to SC CO_2_ influence and release of the enzyme into the cell suspension. It is obvious that α-amylase from *P. triangularis* is less sensitive to SC CO_2_ than α-amylase from *T. salinum* and *W. ichityophaga* since its activity remains the same (30 MPa) or even increases (10 MPa) at higher incubation times.

#### Residual Activities of β-Glucosidases

β-Glucosidases are present in bacteria, fungi, plants, and animals. Fungi produced β-glucosidase is an enzyme that breaks down cellulose and works in synergy with endoglucanases and cellobiohydrolases. They split cellobiose into two molecules of glucose, protecting the above mentioned enzymes from the product inhibition effect of cellobiose. Most of the β-glucosidases from bacteria and fungi are mainly intracellular enzymes. Molds secrete their enzymes extracellularly. Most of these microbial enzymes have an optimum pH in an acidic range and are active in a broad range of pH. Also important is the resistance of these enzymes to higher temperature, since some biotechnological processes take place at high temperatures. The temperature range in which the β-glucosidase is active is from 25 to 75°C (Kirsch et al., 2010).

Their application in the conversion of high-cellulose-content biomass to fermentable sugars for the production of fuel ethanol is an intensively studied area. Good β-glucosidase-producer fungi, usable in various biotechnological processes, synthesize these enzymes with high hydrolyzing activity, heat and glucose tolerance, acid resistance, and possible transglycosylase activity (Kirsch et al., 2010).

**Figure 7:**
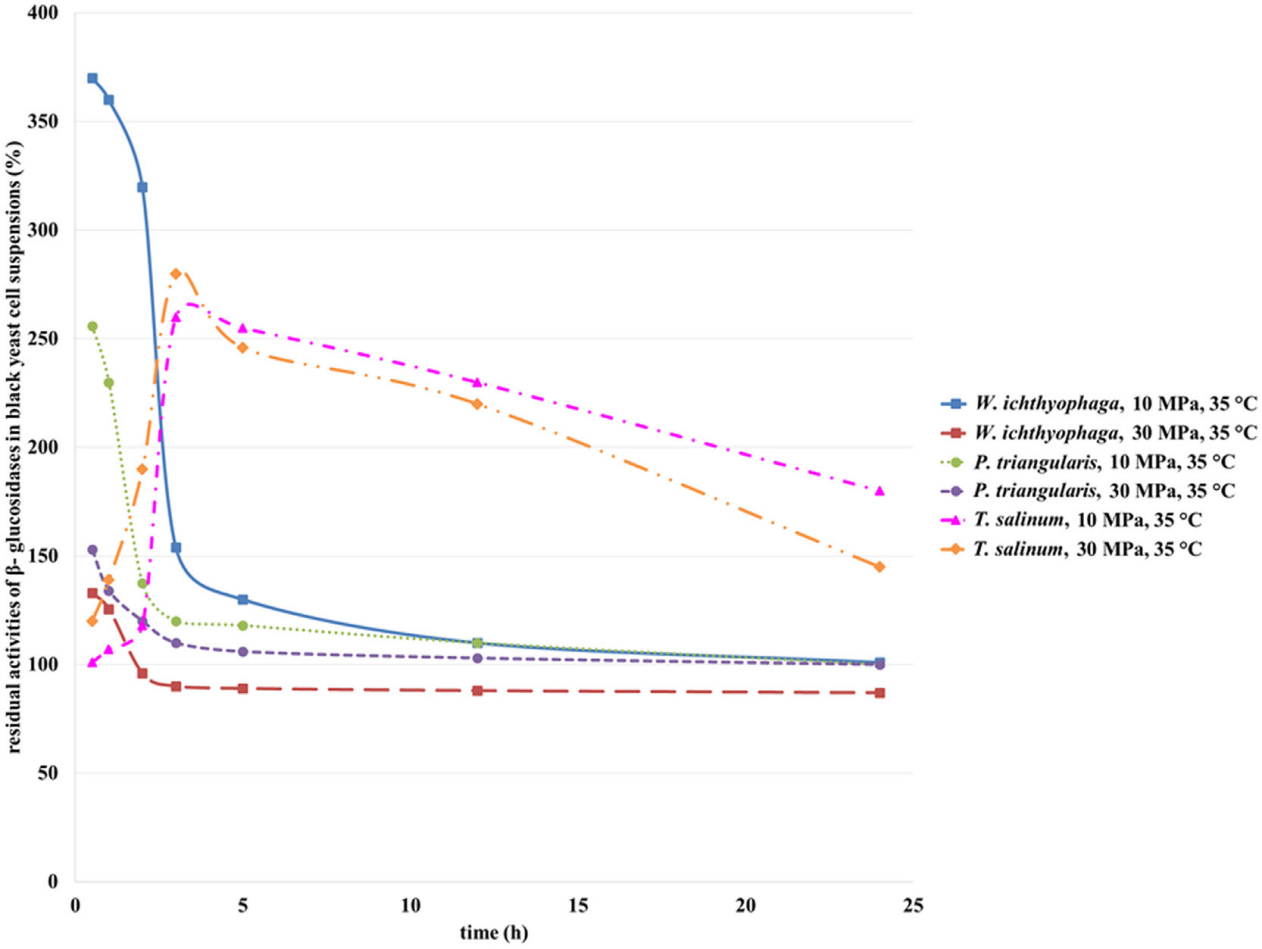
**Residual activities of β-glucosidases from *W. ichthyophaga, P. triangularis*, and *T. salinum* after incubation in SC CO_2_ at 10 and 30 MPa and at 35°C as a function of incubation time**.

β-Glucosidases from all three treated black yeasts are extracellular enzymes (Zalar et al., 2005b) (personal communication, Zalar and Gunde-Cimerman, 2010). The activity of β-glucosidase from black yeast cells before incubation in SC CO_2_ was defined as 100%. As can be seen from the Figure 7, with an increase in incubation time, a decrease in β-glucosidase residual activities from *W. ichthyophaga* and *P. triangularis* were detected. The highest residual activity was detected for β-glucosidase from *W. ichthyophaga* treated in SC CO_2_ at 10 MPa, 35°C for 30 min. With an increase in incubation time, a significant loss in residual activity was observed. After 24 h of incubation of *W. ichthyophaga* cell suspension in SC CO_2_ at 10 MPa and 35°C, β-glucosidase reached the same activity as in untreated cell suspension. When the *W. ichthyophaga* cell suspension was incubated in SC CO_2_ at 30 MPa, already after 2 h only 96% of residual activity was detected. Higher residual activity of β-glucosidase from *P. triangularis* was observed at lower studied pressure (10 MPa), and the highest residual activity was reached when the *P. triangularis* cell suspension was incubated in SC CO_2_ at 10 MPa and 35°C for 30 min.

The residual activity of β-glucosidase in *T. salinum* cell suspension increased with increase in incubation time from 30 min to 3 h. Additional increase in incubation time caused a decrease in its residual activity at both studied pressures. The highest residual activity of β-glucosidase in *T. salinum* cell suspension (280%) was observed at 10 MPa, 35°C, and incubation time, 5 h.

Obviously, the increase in incubation time of studied black yeasts in SC CO_2_ influences the residual activity of β-glucosidase. High pressure together with long treatment time in SC CO_2_ led to deactivation of β-glucosidase.

#### Residual Activities of Proteases

Proteases occur in all organisms, from prokaryotes to eukaryotes and viruses. These enzymes catalyze multitude of reactions from simple digestion of food proteins to highly regulated cascades. Most commercial proteases are derived from microorganisms belonging to the order *Bacillus*. An example of a high alkaline protease activity was detected in *Penicillium chrysogenum*. The enzyme produced by microorganisms occurs in detergents and textile industries. Fungi that occur in natural habitats with variable environmental conditions are important from an industrial point of view, because they produce new metabolites and enzymes with hyperactive catalytic properties (Sethi and Gupta, 2015).

**Figure 8:**
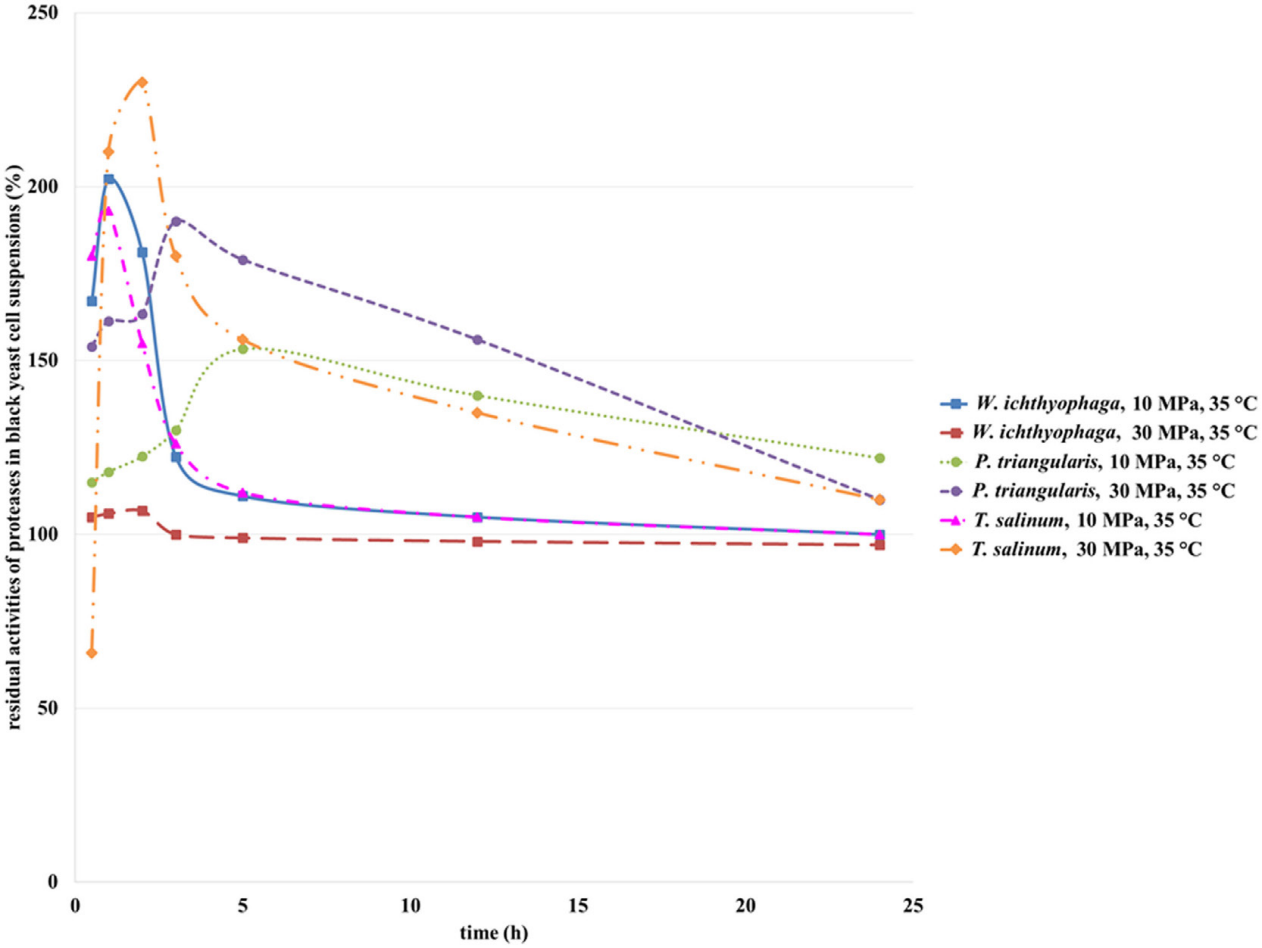
**Residual activities of proteases from *W. ichthyophaga*, *P. triangularis*, and *T. salinum* after incubation in SC CO_2_ at 10 and 30 MPa and at 35°C as a function of incubation time**.

Proteases in cell suspensions of *T. salinum* (Zalar et al., 2005b) and *W. ichthyophaga* are intracellular enzymes, while the protease from *P. triangularis* cells is an extracellular enzyme (personal communication, Zalar and Gunde-Cimerman, 2010). With an increase in incubation time of *W. ichthyophaga* cell suspension in SC CO_2_ at 10 MPa and 35°C up to half an hour, an increase in residual activity of protease was detected (Figure 8). With additional increase in incubation time, a decrease in residual activity was observed. No significant changes in activity of proteinase from *W. ichthyophaga* cell suspensions at higher pressure (30 MPa) were detected.

The residual activity of protease from *T. salinum* cell suspension increased with higher incubation time in SC CO_2_ up to 2 h at 30 MPa and up to 1 h at 10 MPa and then by prolongation of incubation time in SC CO_2_, a decrease in residual activities of proteases was observed. Obviously, high pressure and longer incubation time influenced this enzyme and reduced its activity.

Due to earlier inactivation of *T. salinum* cells during incubation in SC CO_2_ and the consequent earlier opening of cells, the residual activity of protease from *T. salinum* after 2 h of incubation in SC CO_2_ reached its maximum (230%). Further, with increase in incubation time, deactivation of enzyme occurred. In contrast to *T. salinum*, the inactivation of *W. ichthyophaga* cells occured later, but with the lengthening of incubation time in SC CO_2_ deactivation of protease appeared, as well.

In Table S2 in Supplementary Material, the maximum residual activities of selected enzymes in black yeasts cell suspensions at different conditions are presented.

As could be seen from the Table S2 in Supplementary Material, for all selected enzymes in black yeasts suspensions, very high maximum residual activities after incubation in SC CO_2_ were detected. In the cases of cellulase, α-amylase, and protease (intracellular enzymes), high residual activities of enzymes were probably reached due to SC CO_2_ influence on cell opening and release of the enzyme into the cell suspension. The highest maximum residual activities were detected at higher pressure and shorter incubation time. Obviously, prolongation of incubation time in SC CO_2_ has a negative influence on residual activity of enzymes, because it causes their deactivation. Since cellulase is an intracellular enzyme of *W. ichthyophaga*, the highest maximum residual activity of cellulase in *W. ichthyophaga* cell suspension is attributed to a large quantity of extracted cellulase from the cells of *W. ichthyophaga*. High pressure and long incubation time in SC CO_2_ have enabled opening of the cells, and subsequently, the higher amount of cellulase was released from the cells.

## Conclusion

Enzymes are favorable catalysts for the development of environmentally benign industrial process. Therefore, the use of microbial enzymes is a key step in the development of industrial bioprocesses. Using whole cells as a source of biocatalysts generate less waste and allow the application of industrial processes in milder conditions. Furthermore, biocatalysis using whole cell eliminates the need for enzyme purification and immobilization. The advantage of using SC CO_2_ in biocatalysis is in the fact that it can act as a medium for opening the cells and carrying out the biocatalytical reaction at the same time.

Influence of pressure and incubation time of SC CO_2_ on the viability of black yeast cells and enzyme activities of cellulase, β-glucosidase, protease, and α-amylase was studied. From the black yeast cells during the SC CO_2_ treatment, a significantly high amount of proteins was released. Based on obtained results, it can be concluded that various enzymes in their active form can be released from cells, which were previously incubated in SC CO_2_. Thus obtained enzymes from microbial cells could be used for further applications, e.g., for the cascade reactions, where for the synthesis of a particular product a number of different enzymes are required.

## Author Contributions

MČ carried out all the experiments and wrote the manuscript. MP participated in manuscript writing, data interpretation and corrected the manuscript. ŽK made short literature review. ML participated in study conception, data interpretation, and corrected the manuscript. All authors have read and approved the final manuscript.

## Conflict of Interest Statement

The authors declare that the research was conducted in the absence of any commercial or financial relationships that could be construed as a potential conflict of interest.

## References

[B1] Bradford M. M. (1976). A rapid and sensitive method for the quantitation of microgram quantities of protein utilizing the principle of protein-dye binding. Anal. Biochem. 72, 248–254. 10.1016/0003-2697(76)90527-3 942051

[B2] Ceni G. Fernandes Silva M. Valério C. Jr. Cansian L. Oliveira R. Dalla Rosa J. V. (2016). Continuous inactivation of alkaline phosphatase and *Escherichia coli* in milk using compressed carbon dioxide as inactivating agent. J. CO2 Util. 13, 24–28. 10.1016/j.jcou.2015.11.003

[B3] Darani K. K. Mozafari M. R. (2009). Supercritical fluids technology in bioprocess industries: a review. J. Biochem. Tech. 2, 144–152.

[B4] Debs-Louka E. Louka N. Abraham G. Chabot V. Allaf K. (1999). Effect of compressed carbon dioxide on microbial cell viability. Appl. Environ. Microbiol. 65, 626–631. 9925592 10.1128/aem.65.2.626-631.1999 PMC91071

[B5] Erkmen O. (2003). Mathematical modeling of *Saccharomyces cerevisiae* inactivation under high-pressure carbon dioxide. Nahrung 47, 176–180. 10.1002/food.200390041 12866619

[B6] Garcia-Gonzalez L. Geeraerd A. H. Spilimbergo S. Elst K. Van Ginneken L. Debevere J. (2007). High pressure carbon dioxide inactivation of microorganisms in foods: the past, the present and the future. Int. J. Food Microbiol. 117, 1–28. 10.1016/j.ijfoodmicro.2007.02.018 17475355

[B7] Geciova J. Bury D. Jelen P. (2002). Methods for disruption of microbial cells for potential use in the dairy industry-a review. Int. Dairy J. 12, 541–553. 10.1016/S0958-6946(02)00038-9

[B8] Gunde-Cimerman N. Zalar P. de Hoog S. Plemenitas A. (2000). Hypersaline waters in salterns – natural ecological niches for halophilic black yeasts. FEMS Microbiol. Ecol. 32, 235–240. 10.1016/S0168-6496(00)00032-5 10858582

[B9] Hong S. Pyun Y. (2001). Membrane damage and enzyme inactivation of *Lactobacillus plantarum* by high pressure CO_2_ treatment. Int. J. Food Microbiol. 63, 19–28. 10.1016/S0168-1605(00)00393-7 11205950

[B10] Ji H. Zhang L. Liu S. Qu X. Zhang C. Gao J. (2012). Optimization of microbial inactivation of shrimp by dense phase carbon dioxide. Int. J. Food Microbiol. 156, 44–49. 10.1016/j.ijfoodmicro.2012.02.020 22424934

[B11] Karthikeyan V. Santosh S. W. (2009). Isolation and partial characterization of bacteriocin produced from *Lactobacillus plantarum*. Afr. J. Microbiol. Res. 3, 233–239.

[B12] Krisch J. Takó M. Papp T. Vágvölgyi C. (2010). “Characteristics and potential use of β-glucosidases from Zygomycetes”, in current research, technology and education topics in applied microbiology and microbial biotechnology, ed. Méndez-Vilas A. Formatex Research Center, Vol. 2 891–896.

[B13] Kogej T. Gorbushina A. A. Gunde-Cimerman N. (2006). Hypersaline conditions induce changes in cell-wall melanization and colony structure in a halophilic and a xerophilic black yeast species of the genus *Trimmatostroma*. Mycol. Res. 110, 713–724. 10.1016/j.mycres.2006.01.014 16765585

[B14] Kuhad C. R. Gupta R. Singh A. (2011). Microbial cellulases and their industrial applications. Enzyme Res. 2011, 1–11. 10.4061/2011/280696 PMC3168787 21912738

[B15] Kutty N. S. (2009). Marine Yeasts from the Slope Sediments of Arabian Sea and Bay of Bengal. Dissertation/Master’s thesis, Cochin University of Science and Technology, Cochin.

[B16] Manas P. Pagan P. (2005). Microbial inactivation by new technologies of food preservation. J. Appl. Microbiol. 98, 1387–1399. 10.1111/j.1365-2672.2005.02561.x 15916651

[B17] Mun S. Hahn J. S. Lee Y. W. Yoon J. (2011). Inactivation behavior of *Pseudomonas aeruginosa* by supercritical N_2_O compared to supercritical CO_2_. Int. J. Food Microbiol. 144, 372–378. 10.1016/j.ijfoodmicro.2010.10.022 21078533

[B18] Ortuño C. Martínez-Pastor M. T. Mulet A. Benedito J. (2012). Supercritical carbon dioxide inactivation of *Escherichia coli* and *Saccharomyces cerevisiae* in different growth stages. J. Supercrit. Fluids 63, 8–15. 10.1016/j.supflu.2011.12.022

[B19] Raghukumar C. (2012). Biology of Marine Fungi. Berlin, Heidelberg: Springer-Verlag.

[B20] Rosa C. A. Peter G. (2006). Biodiversity and Ecophysiology of Yeasts. Berlin, Heidelberg: Springer-Verlag.

[B21] Senyay-Oncel D. Yesil-Celiktas O. (2011). Activity and stability enhancement of α-amylase treated with sub- and supercritical carbon dioxide. J. Biosci. Bioeng. 112, 435–440. 10.1016/j.jbiosc.2011.07.012 21824817

[B22] Sethi S. Gupta S. (2015). Optimization of protease production from fungi isolated from soil. Int. J. Appl. Biol. Pharm. 6, 149–155.

[B23] de Souza P. M. de Oliveira Magalhães P. (2010). Application of microbial α-amylase in industry – a review. Braz. J. Microbiol. 41, 850–861. 10.1590/S1517-83822010000400004 24031565 PMC3769773

[B24] Spilimbergo S. Bertucco A. Lauro F. M. Bertoloni G. (2003). Inactivation of *Bacillus subtilis* spores by supercritical CO_2_ treatment. Innov. Food Sci. Emerg. Technol. 4, 161–165. 10.1016/S1466-8564(02)00089-9

[B25] Sterflinger K. (2006). Black Yeast and Meristematic Fungi: Ecology, Diversity and Identification, Vol. 20 Germany: Berlin Heidelberg: Springer, 501–514.

[B26] Sundarram A. Thirupathihalli P. K. M. (2014). α-amylase production and applications: a review. Appl. Environ. Microbiol. 2, 166–175. 10.12691/jaem-2-4-10

[B27] Wimmer Z. Zarevúcka M. (2010). A review on the effects of supercritical carbon dioxide on enzyme activity. Int. J. Mol. Sci. 11, 233–253. 10.3390/ijms11010233 20162013 PMC2821001

[B28] Zajc J. Kogej T. Ramos J. Galinski E. A. Gunde-Cimerman N. (2014). The osmoadaptation strategy of the most halophilic fungus *Wallemia ichthyophaga*, growing optimally at salinities above 15% NaCl. Appl. Environ. Microbiol. 80, 247–256. 10.1128/aem.02702-13 24162565 PMC3911034

[B29] Zalar P. de Hoog G. S. Gunde-Cimerman N. (1999). *Trimmatostroma salinum*, a new species from hypersaline water. Stud. Mycol. 43, 57–62.

[B30] Zalar P. de Hoog G. S. Schroers H. J. Frank J. M. Gunde-Cimerman N. (2005a). Taxonomy and phylogeny of the xerophilic genus *Wallemia* (*Wallemiomycetes* and *Wallemiales*, cl. et ord. nov.). Antonie Van Leeuwenhoek 87, 311–328. 10.1007/s10482-004-6783-x 15928984

[B31] Zalar P. Kocuvan M. A. Plemenitaš A. Gunde-Cimerman N. (2005b). Halophilic black yeasts colonize wood immersed in hypersaline water. Botanica Marina 48, 323–326. 10.1515/bot.2005.042

