# Supplementary material for: Use of Non-Conventional Cell Disruption Method for Extraction of Proteins from Black Yeasts
Source: Front Bioeng Biotechnol. 2016 Apr 15;4:33. doi: 10.3389/fbioe.2016.00033 (PMC4831980; doi:10.3389/fbioe.2016.00033)
Supplement: Supplementary file 1 [file Data_Sheet_1.docx]

Supplementary Material

**Use of non-conventional cell disruption method for extraction of proteins from Black yeast**

**Maja Čolnik, Mateja Primožič, Željko Knez, Maja Leitgeb***

Laboratory for Separation Processes and Product Design, Faculty of Chemistry and Chemical Engineering, University of Maribor, Maribor, Slovenia

*** Correspondence:**Prof. Dr. Maja Leitgeb
[maja.leitgeb@um.si](mailto:maja.leitgeb@um.si)

## Supplementary Tables

**Supplementary Table 1.** The physical properties of CO_2_.

|  | ^a)^Density (g/mL) | ^a)^Viscosity (Pas) | Solubility of water | ^b)^pH before | ^b)^pH after |
| --- | --- | --- | --- | --- | --- |
| 10 MPa, 35 °C | 0.71 | 5.77 x 10^-5^ | 4.05 x 10^-5^ | 5.8 | 4.6 |
| 30 MPa, 35 °C | 0.93 | 9.89 x 10 ^-5^ | 6.25 x 10^-5^ | 5.8 | 4.4 |

^a)^ Density and viscosity at 10 MPa and 30 MPa at 35 °C achieved in <http://webbook.nist.gov/chemistry/>. NIST Standard Reference Database Number 69,

^b)^ pH was measured in the black yeast suspensions before and after incubation in SC CO_2_ at 10 and 30 MPa and 35 °C.

**Supplementary Table 2.** The maximum residual activity in black yeasts cell suspensions (%) at different conditions.

| Enzymes | The maximum residual activity in black yeasts cell suspensions (%) | Pressures and incubation times in SC CO_2_ | Black yeasts cell suspensions |
| --- | --- | --- | --- |
| cellulase | 300 | 30 MPa, 24 h | *W. ichthyophaga* cell suspension |
| α-amylase | 400 | 30 MPa, 5 h | *T. salinum* cell suspension |
| β-glucosidase | 370 | 10 MPa, 30 min | *W. ichthyophaga* cell suspension |
| protease | 230 | 30 MPa, 2 h | *T. salinum* cell suspension |
